# Possible Association of Periodontal Disease and Macular Degeneration: A Case-Control Study

**DOI:** 10.3390/dj9010001

**Published:** 2020-12-22

**Authors:** Federica Di Spirito, Michele La Rocca, Maddalena De Bernardo, Nicola Rosa, Carolina Sbordone, Ludovico Sbordone

**Affiliations:** 1 Department of Medicine, Surgery and Dentistry “Schola Medica Salernitana”, University of Salerno, Via S. Allende, 84081 Baronissi, Italy; mdebernardo@unisa.it (M.D.B.); nrosa@unisa.it (N.R.); lsbordone@unisa.it (L.S.); 2 Department of Economics and Statistics, University of Salerno, Via Giovanni Paolo II 132, 84084 Fisciano, Italy; larocca@unisa.it; 3 Department of Radiology, School of Medicine, University of Molise, Via Giovanni Paolo II, Contrada “Tappino”, 86100 Campobasso, Italy; carolina.84@hotmail.it

**Keywords:** ARMD, macular degeneration, periodontitis

## Abstract

Oral pathogens have been identified in bioptic specimens from Age-Related Macular Degeneration (ARMD) patients, and alveolar bone loss has been related to ARMD. Therefore, the possible association between ARMD and periodontal disease was investigated in the present case-control study, evaluating clinical and radiographic periodontal parameters, primarily, in cases vs. controls and, secondarily, in relation to ARMD risk factors, in cases, to highlight a possible pathogenic link between the disorders. Forty ARMD cases and 40 non-ARMD controls, matched for age (±3 years) and gender and homogeneous for ARMD risk factors, therefore comparable, underwent full-mouth periodontal charting, panoramic radiograph, and medical data, including ARMD risk factors, collection. Statistical analysis was conducted using the language R. Comparisons between groups were made using both traditional *t*-tests and Yuen’s test with bootstrap calibration. Enrolled subjects were ≥55 years old, and 50 females and 30 males were equally distributed among the two groups. No statistically significant difference was found in clinical and radiographic periodontal parameters in cases vs. controls. In the case group, no differences were found when relating the periodontal parameters to ARMD risk factors, except for Clinical Attachment Level values that were statistically significantly higher in hypertensive ARMD subjects. A possible association between periodontal disease and ARMD may be hypothesized in hypertensive ARMD subjects, with hypertension as a possible pathogenic link between the disorders.

## 1. Introduction

Age-Related Macular Degeneration (ARMD) is a progressive degenerative vision-threatening ocular disease in the elderly. It represents the late stage of a group of degenerative changes in the macula, the central region of the retina and the choroid, collectively called age-related maculopathy [1].

ARMD is characterized by the appearance of drusen in the macula, followed by geographic atrophy or by choroidal neovascularization [2], and affects, primarily, the retinal pigment epithelial cells and, secondarily, the photoreceptors, leading to disturbances or partial loss of central vision and legal blindness [1].

The etiology of ARMD is still not well known, but, together with age-dependence, a complex interaction between genetic and environmental factors appears to be responsible for the onset of the disease [3,4]. The pathogenesis is also not yet completely understood, but the evidence that initial ARMD signs include retinal pigment epithelial changes, resembling Alzheimer disease and atherosclerosis [5], may suggest common etiopathogenic pathways and may be due to shared risk factors, such as age, low antioxidant levels [6], high Body Mass Index (BMI) [7,8], cigarette smoking [9], hypertension, and systemic inflammation [10]. Other established ARMD risk factors are gender, ethnicity, diabetes, abnormal plasma total cholesterol, High-Density Lipoproteins (HDL), and Low-Density Lipoproteins (LDL) levels [3,4].

ARMD has recently been found independently associated, in patients aged 40 to 60 years, with periodontal disease [10,11].

Periodontal disease is a common multifactorial, inflammatory, microbially-associated disease that frequently develops in adult age, characterized by alveolar bone loss and periodontal tissue destruction, leading to tooth loss [12,13,14].

Recently, many researchers have reported that periodontal disease may be associated with various systemic diseases, comprising pulmonary and cardiovascular ones along with diabetes, albuminuria, preterm birth, chronic obstructive, obesity, as well as with solid tumors, including colorectal cancer [15,16], and that periodontal pathogens may act either directly, infiltrating periodontal damaged tissue, entering systemic circulation, and leading to an inflammatory response in other organs, or indirectly via endotoxin production [11,15,17].

In particular, periodontal disease has been found associated with atherosclerosis, as well as atherosclerosis with ARMD [10]; moreover, ARMD has been related to alveolar bone loss [5], and oral pathogens have been identified in bioptic specimens from ARMD patients [10]. These findings may suggest the existence of a possible etiopathogenic link between ARMD and periodontal disease.

Since ARMD is one of the most common causes of blindness in elderly populations in industrialized countries [11,18], andits prevalence is predicted to continuously increase because of the aging population [19], etiopathogenesis identification and risk factors management of this condition appear crucial [10]. Therefore, highlighting a possible role a periodontal disease in ARMD etiopathogenesis may, beyond improving knowledge about oral and general health inter-relationship, contribute to a multi-disciplinary integrated approach to ARMD patients.

Thus, the present case-control study aimed to investigate, primarily, the possible association between periodontitis and ARMD, comparing periodontal parameters and alveolar bone loss in ARMD vs. matched non-ARMD subjects, and, secondarily, the possible pathogenic link between the disorders in ARMD subjects, evaluating cases periodontal clinical and radiographic parameters in relation to ARMD risk factors, such as smoking habit, cholesterol, HDL and LDL levels, BMI, diabetes, and hypertension.

## 2. Materials and Methods

### 2.1. Study Design and Sample

The present observational case-control study was approved by the local Ethical Committee (Ethics Committee Azienda Ospedaliero-Universitaria San Giovanni di Dio e Ruggid’Aragona approved by Protocol n. 34/2013 on 6 May 2013, confirmed by the resolution # 776 6 August 2014) and conducted according to The Code of Ethics of the World Medical Association (Declaration of Helsinki).

Cases were 40 subjects diagnosed with ARMD at the Ophthalmology Clinic of the Azienda Ospedaliero-Universitaria San Giovanni di Dio e Ruggi d’Aragona, Salerno, Italy, and attending the Complex Operating Unit of Odontostomatology of the Azienda Ospedaliero-Universitaria San Giovanni di Dio e Ruggi d’Aragona, Salerno, Italy, between May 2014 and March 2019. Controls were 40 non-ARMD subjects seeking a routine oral examination at the same dental unit, in the same time period, matched for age (±3 years) and gender to ARMD subjects and homogeneous in relation to BMI, blood pressure, hypertension, and total cholesterol with cases, in order to be comparable.

The inclusion criteria were: age ≥ 18 years, teeth ≥ 3. The exclusion criteria were: age < 18 years, pregnancy, neoplastic disease, edentulism, oral and systemic infections, medication-related osteonecrosis of the jaws, periodontal treatment, antibiotic or corticosteroid therapy in the last 3 months. In addition, cases with advanced cataract or other ocular diseases potentially interfering with OCT examination, as well as controls having visual impairment during the medical data collection, were excluded from the study.

All enrolled subjects, cases and controls, who agreed to participate in the study, underwent an oral and periodontal examination, a panoramic radiograph, and a medical data collection; informed written consent was obtained from each one.

### 2.2. ARMD Case Definition and Grading

Preliminary to case enrollment, ARMD clinical diagnosis was made through a complete ophthalmic examination, including measurement of best-corrected visual acuity, according to the ETDRS visual logarithm of the minimum angle of resolution scale; slit-lamp examination; intraocular pressure measurement; fundus examination; a Spectralis SD-OCT system (Heidelberg Engineering, Heidelberg, Germany) to confirm the diagnoses.

All the OCT scans were acquired by the same experienced operator with a Spectralis OCT system (software version 6.0), using the horizontal 30″-line scan EDI mode through the fovea. The line scan images were saved for analysis after 100 frames were averaged, using an automatic real-time imaging value of 100 and the active eye-tracking feature [20].

Cases were then graded according to the National Health and Nutrition Examination Survey Data Examination File (NHANES III) protocol [10].

Ophthalmic examination was not performed on controls.

### 2.3. Periodontal Disease Case Definition and Staging

A complete oral examination with a periodontal full-mouth charting and a panoramic radiograph of both cases and controls was performed at the Complex Operating Unit of Odontostomatology of the Azienda Ospedaliero-Universitaria San Giovanni di Dio e Ruggi d’Aragona, Salerno, Italy.

The total number of teeth was recorded for each patient. Periodontal charting consisted of the assessment of Clinical Attachment Level (CAL) and Periodontal Pocket Depth (PPD) in millimeters, Gingival Index (GI) [21], and Plaque Index (PlI) [22], all registered as six values around each tooth. All measurements were taken under the same conditions, in a dental chair equipped with professional light, by a single expert and blinded calibrated operator, using a University North Carolina periodontal probe (PCP UNC 15 Probe, Hu-Friedy, Chicago, IL, USA). Tooth mobility and class furcation were also recorded. Full Mouth Plaque Score (FMPS%) and Full Mouth Bleeding Score (FMBS%) [23] were calculated.

Panoramic X-rays were scored and assigned to Radiographic Bone Loss (RBL) stages, as per Tonetti et al. [24], and also to alveolar bone loss classes, as previously described by Karesvuo et al. [5], by a single-blinded examiner not involved in the periodontal charting.

Periodontitis case definition was performed for both cases and controls, according to the 2017 classification of periodontal and peri-implant diseases and conditions [24].

### 2.4. Medical Data Collection

Data, including established ARMD risk factors, were collected from all enrolled subjects in order to match cases and controls, regarding age and gender; weight height and BMI; smoking habit; previously diagnosed hypertension, defined as already having a prescription for antihypertensive medication; previously diagnosed diabetes and glycosylated hemoglobin in diabetic subjects; total cholesterol, HDL, LDL; triglycerides and C-reactive protein; reported eye diseases, neoplasms, infections; antibiotic or corticosteroid therapy in the last 3 months; periodontal treatments in the last 3 months [3,4,25].

Current blood pressure values, registered on the day of the clinical examination and consistent with the ones reported by the referring physician, were recorded for each subject.

### 2.5. Data Management and Statistical Analysis

Data management and statistical analysis were implemented by using the statistical language R (version 3.5.3). The package tidy verse was used for data wrangling and visualization, while the package WRS2 was used for robust statistical inference. Comparisons between groups were made by using the standard *t*-test. Yuen’s test for trimmed means with bootstrap calibration (using 1999 bootstrap runs) was employed to check the stability of the results in the presence of outliers. The test is resistant to outliers (with a reasonable percentage of trimming) and allows for heteroscedasticity [26]. Power analysis was handled using the R package pwr for standard *t*-test and by using the approach proposed by LuhandGuo [27] for a trimmed *t*-test with unequal variances (heteroscedasticity).

The sample size was calculated, according to Cohen’s scale for the quantitative variables, considering that to detect a standardized difference of 0.8 with a power of 0.80 and with a level equal to 0.05, a sample size of 26 cases and 26 controls is required; thus, with the sample size of the present study, numbering 40 cases and 40 controls, the statistical power increases to 0.94, and, for the categorical variables, considering that to detect a standardized difference of 0.5, a sample size of32 cases and 32 controls is required; therefore, with the current 40 cases and 40 controls, a statistical power equal to 0.88 can be achieved.

## 3. Results

### 3.1. Sample Characteristics

A total of 80 subjects were enrolled—40 ARMD cases and 40 non-ARMD controls. There were no differences in age, gender, or ARMD risk factors between cases and controls. All participants were ≥55 years old, previously matched for age (±3 years), and with a mean age of 75.8 years for the case group and 71.2 years for the control group, respectively. The 50 females and 30 males included in the study, previously matched also for gender, were equally distributed among the two groups. Enrolled subjects were homogeneous in relation to ARMD risk factors.

**Figure 1:**
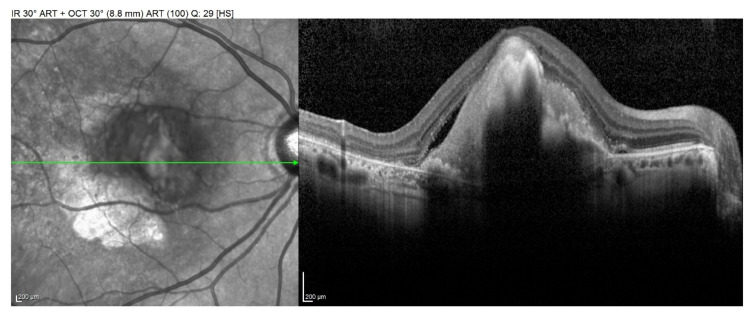
Structural Optical Coherence Tomography (OCT) scan showing a neovascular Age-Related Macular Degeneration (ARMD).

**Figure 2:**
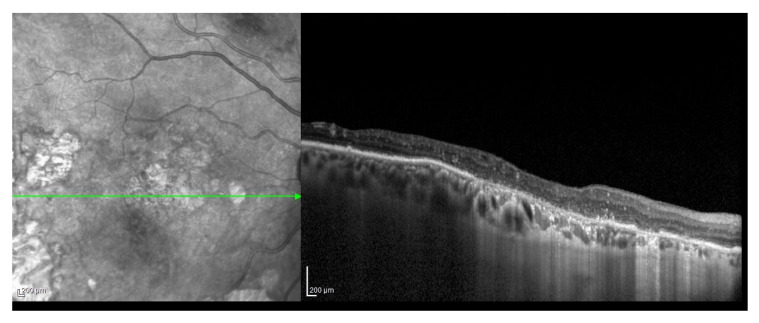
Structural OCT scan showing a neovascular ARMD.

Among the cases, 28 patients were classified as early, non-exudative ARMD, whereas 12 subjects were classified as late ARMD, consisting of either neovascular ARMD (eight subjects) or geographic atrophy (four subjects), illustrated, for example, in Figure 1 and Figure 2, respectively.

### 3.2. Dental and Periodontal Data

Mean teeth number, Mean CAL (mCAL), Mean PPD (mPPD), FMBS%, and FMPS% values are shown for both cases and controls in Table 1. Mean values for CAL and PPD were computed as averages for each patient’s tooth and then for every single patient. Given that the subjects had different teeth numbers, the measures were heteroscedastic; this statistical aspect was taken into account in all testing procedures, as reported in the Data Management and Statistical Analysis section.

Interdental CAL values at the site of greatest loss, as per Tonetti et al. (2018), are reported in Table 2 for both cases and controls; 95% of subjects showed overall high circumferential CAL (≥5 mm) values. Alveolar bone loss stages distribution for both case and control groups, as per Tonetti et al. [24] and as per Karesvuo et al. [5], is presented in Table 3. All cases, as well as all controls, were affected by generalized (≥30% of teeth involved) periodontitis.

#### 3.2.1. Periodontal Parameters in Cases vs. Controls

**Figure 3:**
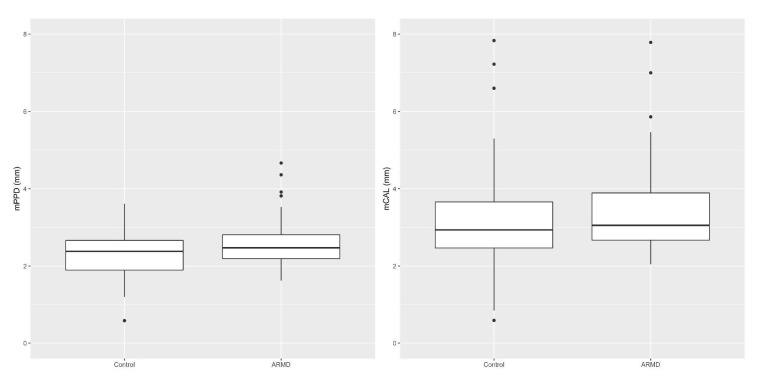
Comparison of Mean PPD (mPPD) and Mean CAL (mCAL) values in cases vs. controls (non-statistically significant at α = 0.05).

No statistically significant difference was found between case and control groups regarding teeth number, PPD and CAL values, or radiographic bone loss stage distribution (Figure 3).

Almost all subjects (≥85%) showed a radiographic bone loss extending to the middle or apical third of the root, as per Tonetti et al. [24], and most of the subjects (>60%) were addressed to class 1 and 2 of bone loss, as per Karesvuo et al. [5] (Table 3).

#### 3.2.2. Periodontal Parameters in Relation to ARMD Risk Factors in Cases

Restricting the analysis to cases, the possible relationship of teeth number, PPD and CAL values, and radiographic bone loss to gender, smoking habit, BMI, cholesterol levels, and diabetes was investigated, and no statistical significance was found.

No statistically significant difference was found when observing teeth number in relation to blood pressure measurements distribution in cases.

**Figure 4:**
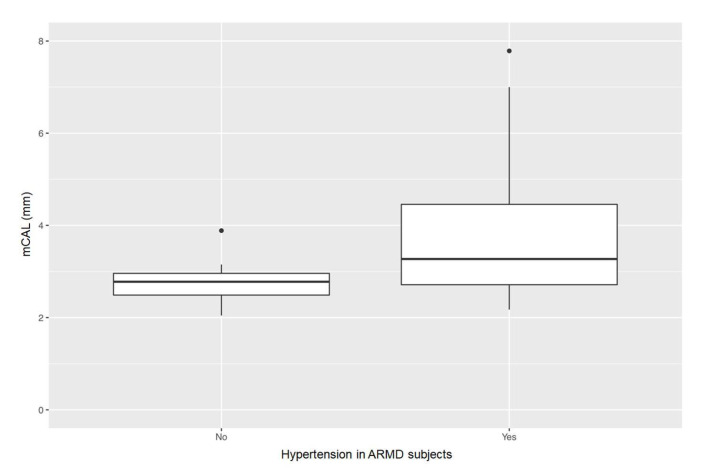
Comparison of mCAL values between ARMD subjects affected or not by hypertension (statistically significant at α = 0.05).

Neither a statistical significance was found on analyzing teeth number, PPD values, nor radiographic bone loss stage between cases affected by hypertension and non-hypertensive ones. CAL values, instead, were significantly statistically higher in cases affected by hypertension compared to the non-hypertensive ones (*p*-value = 0.005; mean difference: −0.980. 95% confidence interval: −1.577 −0.383), as shown in Figure 4.

## 4. Discussion

The possible association between periodontitis and ARMD in humans was investigated in the present case-control study, comparing clinical and radiographic periodontal parameters in cases vs. controls, and evaluating their relation with ARMD risks factors (i.e., smoking habit, cholesterol, HDL and LDL levels, BMI, diabetes, and hypertension), in the case group, to potentially highlight a possible pathogenic link between the disorders.

Both case and control groups, prior matched for age and gender, were homogeneous in relation to BMI, blood pressure, cholesterol, and triglycerides, so being comparable.

In the present survey, all the cases were ≥55 years of age, in agreement with Jonas et al. [28], who reported an age range of 45–85 years for ARMD clinical onset, with a marked increase over 75 years of age in all ethnicities.

### 4.1. Periodontal Parameters in Cases vs. Controls

On evaluating clinical periodontal parameters (CAL and PPD), no statistically significant differences were found when comparing cases and controls, and periodontal status was similarly compromised between ARMD and non-ARMD subjects. The poor control of the local etiologic factors, as shown by FMPS and FMBS values, along with the overall high extent and severity of periodontitis found in the present study, in both cases and controls, may explain such results. Moreover, it may be speculated that the higher FMPS% and FMBS% found in cases may be possibly related to the reduced tooth brushing efficacy secondary to the visual impairment.

In contrast with our finding, Brzozowska et al. [29], studying oral conditions as causative or risk factors for ARMD, reported a high prevalence of dental and periodontal lesions in ARMD patients and suggested a possible association between periodontal conditions and susceptibility to ARMD onset; however, this was a retrospective study and without controls, in contrast to the present survey.

The association between periodontal disease and ARMD was also described in Caucasian patients between 40 and 60 years of age, but not in older ones (>60 years of age), by Wagley et al. [10], applying a periodontal disease case definition proposed by the U.S. National Institute of Dental and Craniofacial Research, published, as referenced, in an informative booklet. Similar results were later found in an Asian ARMD population of the same age (≥40 y.o.) by Shin et al. [11], applying the WHO Community Periodontal Index for periodontal disease occurrence. Currently presented results may be in contrast with these authors’ findings, in part because of a different case definition of periodontitis, as the present study used the recommended 2017 classification of periodontal and peri-implant diseases and conditions [24]—more recent and widely accepted. Additional discordance between our findings and those of Wagley et al. [10] and Shin et al. [11] may be explained by the higher age range (≥55 years) found in this study, in the cases as well as in the controls matched for age (±3 years). Indeed, Shin et al. proposed that concurrently to genetic predisposition, which is a well-known ARMD etiologic factor, the inflammatory pathogenic effect, also related to periodontal disease, might be more evident in middle-age ARMD patients than in older ones, while age-related factors, such as oxidative stress and degenerative changes, may be more pronounced in patients over 60 years of age [11]. In this perspective, the inflammatory pathogenic effect, common to both ARMD and periodontal disease, might be partially hidden, in the present study, by the older age of both groups, cases and controls.

In the present survey, radiographic periodontal parameters (bone loss) were evaluated in the case and in control subjects as per Tonetti et al. [24], and no statistically significant differences were found between the two groups. In order to compare present findings with the ones previously reported by Karesvuo et al. [5], alveolar bone loss was radiographically scored also according to the classification proposed by these authors, and, in addition, no differences were found between cases and controls. Conversely, Karesvuo et al. [5], according to their classification, reported a more severe bone loss in ARMD subjects when compared to the controls. Those conflicting findings may be due to the fact that in the Karesvuo et al. study [5], the control group was younger than the ARMD group, whereas, in the current study, the controls were matched for age (±3 years) to cases. Furthermore, from the data on radiographic bone loss reported by Karesvuo et al. [5,30], it might be inferred that the patient population described in that study might have substantially healthier periodontal conditions overall compared with the groups currently observed, possibly explained by the presence of less local etiologic factors. Unfortunately, this can only be speculated since Karesvuo et al. [5] did not report data on the evaluation of plaque accumulation or gingival inflammation. The current survey revealed the definite presence of local etiologic factors and gingival inflammation in both case and control groups, as indicated by FMPS% and FMBS% reported in Table 1, which may hide the possible effect of other risk factors.

### 4.2. Periodontal Parameters in Relation to ARMD Risk Factors in Cases

In the present study, restricting the analysis to the case group to evaluate periodontal parameters in relation to ARMD risk factors in cases, teeth number, CAL and PPD values, and radiographic bone level were challenged against other putative risk factors associated with ARMD, such as smoking habit, cholesterol, HDL and LDL levels, BMI, diabetes, and hypertension.

Only hypertensive cases showed statistically significant higher CAL values compared to non-hypertensive ones. Noteworthy, hypertension has been found to be associated with moderate rather than a severe periodontal disease, classified according to the older proposal of the American Academy of Periodontology and the Centers of Disease Control [31]; conversely, in the present study, ARMD subjects were mostly affected by stage III and IV periodontitis [24]. However, other studies even failed to associate the periodontal disease with hypertension [32]. Moreover, hypertension has been recognized as a risk factor for both cardiovascular disease and ARMD, previously reported as separately associated [11,33,34].

The present study failed to identify statistically significant differences in PPD values between hypertensive and non-hypertensive cases.

Neither found statistically significant differences in teeth number when comparing hypertensive and non-hypertensive cases. This is also a particular finding regarding only ARMD subjects since, previously, Völzke et al. [35] had reported an association between a reduced number of teeth and an increased risk of cerebrovascular and/or cardiovascular diseases, and Taguchi et al. [36] reported, in post-menopausal women, an association between the reduced number of teeth and a higher risk of hypertension. It is possible that a reduced number of teeth may be associated with hypertension either because of the systemic pro-inflammatory effect induced by periodontal disease [32,37] or as a result of incorrect dietary habits secondary to a reduced number of teeth, as initially proposed by De Stefano et al. [38] and Appel et al. [39] and later confirmed by Lowe et al. [40] and Zhu and Hollis [41]. Moreover, Shin et al. [11] reported that the reduction in the number of teeth was directly related to higher systolic blood pressure values; such a relationship was investigated but not found in the present study. Regarding these parameters, the lack of a statistically significant association between the number of teeth and systolic blood pressure and hypertension both in cases and controls may be due to the fact that both groups showed a very reduced number of teeth and diffuse residual ridge resorption [42].

## 5. Conclusions

Even though increasing evidences suggest a possible association between periodontitis and ARMD [10,43,44], ARMD has been previously related to alveolar bone loss, and periodontal pathogens have been identified in bioptic specimens from ARMD patients, in the present study, no significant differences were detected in clinical and radiographic periodontal parameters of cases vs. controls, supporting such an association.

The significant difference in CAL values recorded in hypertensive ARMD subjects when compared to non-hypertensive ones may lead to hypothesize that hypertension may be a pathogenic link between ARMD and periodontal disease.

However, further investigations with larger observational groups are needed to validate this hypothesis since the strict appropriate diagnostic methods herein applied to compare cases and controls have led to a reduced sample size, which may represent a potential weakness of the study, together with the possible selection and confusion biases related to the case-control study design.

A deeper insight into the etiopathogenic mechanisms underlying both ARMD onset and development and the association between macular degeneration and periodontal disease may pave the way for newly introduced patient-centered preventative strategies for the most common cause of blindness in elderly populations in industrialized countries, favoring an integrated multi-disciplinary approach to the elderly patients, thus merging dental specialties and medical knowledge.

## Figures and Tables

**Table 1:** Mean teeth number, Mean CAL, Mean PPD, FMPS%, and FMBS% values distribution in case and control subjects.

	Mean Teeth Number (±SD*)	Mean CAL (±SD*)	Mean PPD (±SD*)	FMBS% (±SD*)	FMPS% (±SD*)
Cases	17.48 (±7.73)	3.48 (±1.28)	2.63 (±0.70)	94.1% (±0.19)	93.9% (±0.18)
Controls	17.33 (±6.68)	3.19 (±1.56)	2.34 (±0.64)	74.4% (±0.37)	81.3% (±0.31)

±SD*: Standard Deviation.

**Table 2:** Interdental CAL values at the site of greatest loss, as per Tonetti et al. (2018), in case and control subjects.

CAL Values at the Site of Greatest Loss	Cases n. (%)	Controls n. (%)
1 to 2 mm	0(0%)	0(0%)
3 to 4 mm	2(5%)	2(5%)
≥5 mm	38 (95%)	38(95%)

**Table 3:** Alveolar bone loss class distribution, as per Karesvuo et al. (2013) and as per Tonetti et al. (2018), in case and control subjects.

Alveolar Bone Loss (Bone Pocket) As per Karesvuoet al. 2013	Cases n. (%)	Controls n. (%)
Class 0 No bone pocket	5 (12.5%)	6 (15%)
Class 1 Bone pocket exceeding the middle third of the root	26 (65%)	24 (60%)
Class 2 Bone pocket exceeding the apical third of the root	9 (22.5%)	10 (25%)
		
Radiographic Bone Loss (RBL) As per Tonetti et al. (2018)	Cases n. (%)	Controls n. (%)
Coronal third (<15%)	2 (5%)	3 (7.5%)
Coronal third (15% to 33%)	3 (7.5%)	3 (7.5%)
Extending to the middle or apical third of the root (>33%)	35 (87.5%)	34 (85%)
